# Lithium Hexamethyldisilazide Endows Li||NCM811 Battery with Superior Performance

**DOI:** 10.1007/s40820-022-00998-z

**Published:** 2023-01-09

**Authors:** Junda Huang, Yaxiong Yang, Yanxia Liu, Jianmin Ma

**Affiliations:** 1 School of Chemistry, Tiangong University, Tianjin, 300387 People’s Republic of China; 2 School of Physics and Electronics, Hunan University, Changsha, 410082 People’s Republic of China; 3 Institute of Science and Technology for New Energy, Xi’an Technological University, Xi’an, 710021 People’s Republic of China

**Keywords:** Lithium metal battery, Electrolyte additive, Cathode electrolyte interphase, Lithium hexamethyldisilazide, Cycling performance

## Abstract

The construction of stable cathode electrolyte interphase (CEI) is the key to improve the NCM811 particle structure and interfacial stability via electrolyte engineering. In He’s work, lithium hexamethyldisilazide (LiHMDS) as the electrolyte additive is proposed to facilitate the generation of stable CEI on NCM811 cathode surface and eliminate H_2_O and HF in the electrolyte at the same time, which boosts the cycling performance of Li||NCM811 battery up to 1000 or 500 cycles with 4.5 V cut-off voltage at 25 or 60 °C.

The increasing demand for energy storage stimulates the extensive research of high energy density batteries [1–3]. Lithium metal battery (LMB) composed by Li anode and LiNi_0.8_Co_0.1_Mn_0.1_O_2_ (NCM811) cathode is one of the most promising candidates while it is applied at high cut-off voltage (i.e., ≥ 4.5 V vs Li/Li^+^). However, the poor cycling stability associated with NCM811 cathode breakage is a critical issue which needs to be addressed urgently [4, 5]. The available way is to construct a robust CEI on NCM811 particle surface as well as eliminate trace H_2_O and HF in electrolyte, which will attack NCM811 particle and Li anode [6, 7]. Thus, finding an effective electrolyte additive to achieve this purpose is highly feasible [8–10].

Recently, He’s group reported an efficient electrolyte additive (lithium hexamethyldisilazide, LiHMDS) to enhance the performance of Li||NCM811 battery and also discussed its working mechanism [11]. As shown in Fig. 1a, the Li||NCM811 battery assembled with baseline electrolyte (BE) (1 M LiPF_6_ in EC, EMC and DMC (1:1:1 by volume), (H_2_O < 20 ppm)) could only maintain 49.13% capacity retention after 1000 cycles with 4.5 V cut-off voltage at 25 °C. In sharp contrast, the Li||NCM811 battery with 0.6 wt% LiHMDS exhibited superior cycling performance. The capacity retention reached up to 73.92% after 1000 cycles. Moreover, the Li||NCM811 battery with LiHMDS could also obtain outstanding cycling stability at 60 °C. The battery with 0.6 wt% LiHMDS could maintain 66.02% capacity retention with high average coulombic efficiency of 99.11% after 500 cycles, while the battery with BE crashed down after about 450 cycles at 60 °C (Fig. 1b). He and his co-workers considered that the remarkable cycling stability of Li||NCM811 cells with 0.6 wt% LiHMDS was attributed to the scavenging ability of H_2_O and HF in the electrolyte by LiHMDS. As shown in Fig. 1c, the concentration of H_2_O in BE is 18.9 ppm, higher than that in the electrolyte with 0.6 wt% LiHMDS (3 ppm). Furthermore, after adding extra 1000 ppm H_2_O into electrolyte, the H_2_O concentration can still maintain a low level of 23.3 ppm with the existent of LiHMDS but surge to 969.2 ppm without LiHMDS. Similarly, the concentration of HF reached 17.58 ppm in BE and increased to 776.45 ppm after the addition of extra 1000 ppm H_2_O (Fig. 1d). However, no HF was detected while the electrolyte contained 0.6 wt% LiHMDS. This confirms the successful elimination of H_2_O and HF in electrolyte by LiHMDS.

**Fig. 1:**
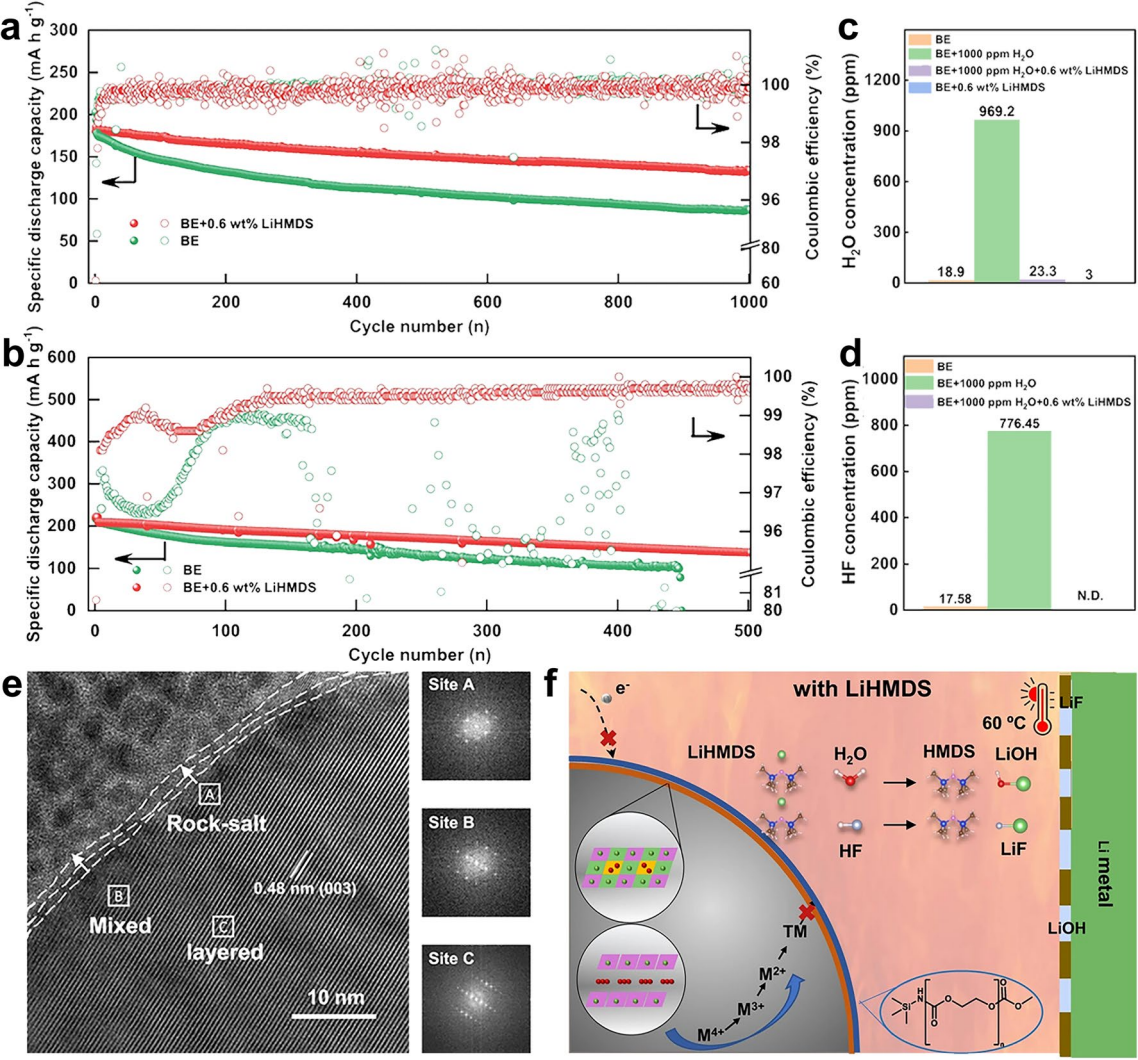
Cycling performance of Li||NCM811 batteries between 3 and 4.5 V with BE and 0.6 wt% LiHMDS-contained electrolyte **a** at 25 °C, 90 mAg^−1^, **b** at 60 °C, 180 mA g^−1^. **c** The concentration of H_2_O in BE, BE + 0.6 wt% LiHMDS, BE + 1000 ppm H_2_O and BE + 0.6 wt% LiHMDS + 1000 ppm H_2_O. **d** The concentration of HF in BE, BE + 1000 ppm H_2_O and BE + 0.6 wt% LiHMDS + 1000 ppm H_2_O. **e** Ex-situ TEM image of NCM811 cathode particle after 100 cycles in the electrolyte with 0.6 wt% LiHMDS at 60 °C. **f** Schematic illustration of LiHMDS with H_2_O and HF in Li||NCM811 cells at 60 °C

In addition, He’s group also verified a robust and uniform cathode electrolyte interphase (CEI) generated on the surface of NCM811 to enhance its structural stability, which was associated with the preferential oxidization of LiHMDS than electrolyte solvents. With the protection of robust CEI, the NCM811 particles avoided harmful reactions with electrolyte and the reduction of high valence Ni^4+^. As a result, the phase transition from layered structure to rock-salt phase was restrained (Fig. 1e), ensuring the stability of NCM811 during continual cycling. The function schematic illustration of LiHMDS in Li||NCM811 battery was demonstrated in Fig. 1f. LiHMDS can capture HF and trace H_2_O in electrolyte to form HMDS and other inorganic products (LiF and LiOH). Besides, LiHMDS can be preferentially oxidized before the electrolyte to build a thin and compact CEI on the surface of NCM811 cathode. Therefore, the superior CEI can inhibit the side reaction of NCM811 with the electrolyte, phase transition from layered structure to rock-salt phase and the dissolution of TM ions from NCM811.

In conclusion, the work from He’s group has given a systematic and comprehensive explanation of LiHMDS as the electrolyte additive, which can bring a remarkable improvement of Li||NCM811 cells performance between 3 and 4.5 V, 25 and 60 ℃. This work provides a novel strategy to preferentially construct a robust CEI on nickel-rich NCM cathodes and eliminate the HF and H_2_O in electrolyte simultaneously for long cycling stability of high-voltage batteries at elevated temperature. It also proves the feasibility of electrolyte additives and guides the further research for high-voltage LMB.
